# Systemic Nicotine Increases Gain and Narrows Receptive Fields in A1 via Integrated Cortical and Subcortical Actions

**DOI:** 10.1523/ENEURO.0192-17.2017

**Published:** 2017-06-22

**Authors:** Caitlin Askew, Irakli Intskirveli, Raju Metherate

**Affiliations:** Department of Neurobiology and Behavior, Center for Hearing Research, University of California, Irvine, CA 92697

**Keywords:** A1, acetylcholine, auditory cortex, mouse, nicotine, receptive field

## Abstract

Nicotine enhances sensory and cognitive processing via actions at nicotinic acetylcholine receptors (nAChRs), yet the precise circuit- and systems-level mechanisms remain unclear. In sensory cortex, nicotinic modulation of receptive fields (RFs) provides a model to probe mechanisms by which nAChRs regulate cortical circuits. Here, we examine RF modulation in mouse primary auditory cortex (A1) using a novel electrophysiological approach: current-source density (CSD) analysis of responses to tone-in-notched-noise (TINN) acoustic stimuli. TINN stimuli consist of a tone at the characteristic frequency (CF) of the recording site embedded within a white noise stimulus filtered to create a spectral “notch” of variable width centered on CF. Systemic nicotine (2.1 mg/kg) enhanced responses to the CF tone and to narrow-notch stimuli, yet reduced the response to wider-notch stimuli, indicating increased response gain within a narrowed RF. Subsequent manipulations showed that modulation of cortical RFs by systemic nicotine reflected effects at several levels in the auditory pathway: nicotine suppressed responses in the auditory midbrain and thalamus, with suppression increasing with spectral distance from CF so that RFs became narrower, and facilitated responses in the thalamocortical pathway, while nicotinic actions within A1 further contributed to both suppression and facilitation. Thus, multiple effects of systemic nicotine integrate along the ascending auditory pathway. These actions at nAChRs in cortical and subcortical circuits, which mimic effects of auditory attention, likely contribute to nicotinic enhancement of sensory and cognitive processing.

## Significance Statement

Nicotinic acetylcholine receptors (nAChRs) are critical for cognitive and sensory processing, and their dysfunction contributes to multiple disorders, including schizophrenia and Alzheimer’s disease. Accordingly, nAChR agonists are being explored as potential therapeutics, yet little is known about the circuit-level mechanisms by which nAChRs enhance cognitive and sensory processing. Here, we probe modulation of auditory receptive fields (RFs) by systemically-administered nicotine and discover that the overall effect in primary auditory cortex results from multiple, integrated effects within the auditory pathway. Our results not only address mechanisms of auditory processing, but, given the similar distribution of nAChRs across cortical areas, may promote an understanding of nicotinic modulation of cortical information processing more generally.

## Introduction

Nicotine is known to enhance cognitive and sensory processing (Rezvani et al., 2002; Levin and Mcclernon, 2006; Warbrick et al., 2012; Gupta and Mittal, 2014), including auditory processing (Knott et al., 2009; Smucny et al., 2015). Nicotine activates nicotinic acetylcholine receptors (nAChRs), which are present throughout the auditory system (Clarke et al., 1985; Morley and Happe, 2000; Dani and Bertrand, 2007; Bieszczad et al., 2012). These nAChRs normally are activated by endogenous ACh, which is a key neuromodulator of cognitive and sensory processes (Himmelheber et al., 2000; Hasselmo and Sarter, 2011; Klinkenberg et al., 2011). Similarly, nicotine is hypothesized to enhance sensory processing through increased attentional filtering, i.e., an increased ability to attend to task-relevant stimuli and ignore distractors (Kassel, 1997; Gilbert et al., 2007; Behler et al., 2015; Smucny et al., 2015). In sensory cortex, activation of nAChRs most often enhances responses evoked by optimal sensory stimuli, but also can produce response suppression to non-optimal stimuli (Liang et al., 2006; Disney et al., 2007; Kawai et al., 2011; Intskirveli and Metherate, 2012). Conversely, loss of cortical nAChRs during aging or disease states is associated with diminished cognitive processing (Whitehouse et al., 1986; Albuquerque et al., 2009), and as a result, nicotine and other nAChR agonists are being considered for therapeutic use (Taly et al., 2009; Hurst et al., 2012; Newhouse et al., 2012). However, beyond its ability to enhance sensory-cognitive function, including sensory-evoked responses, little is known about the circuit-level mechanisms by which nicotine acts. Such an understanding will help to direct development of therapeutic treatments for specific disorders, including central auditory processing disorders.

Here, we investigate physiologic effects of nicotine that are relevant to auditory processing, a broad term encompassing tasks ranging from simple tone detection to speech comprehension (Wallace et al., 2011). In psychoacoustics, a common approach used to examine perceptual filters engaged in auditory processing is to study detection of a tone-in-notched-noise (TINN; Patterson, 1976). That is, a listener is required to detect a tone in the presence of a NN masker, i.e., a white noise stimulus filtered to create a spectral “notch” of variable width centered at the tone frequency. As the notch is progressively narrowed, the width of the hypothetical perceptual filter used to detect the tone is estimated by the notch width at which the tone-detection threshold begins to rise. The physiologic equivalent of a perceptual filter is the frequency receptive field (RF), which traditionally is measured using pure tones (Sutter et al., 1999). However, since TINN stimuli more closely approximate real-life stimuli by activating multiple frequency channels simultaneously, TINN-evoked electrophysiological responses may be more informative for understanding auditory processing. Additionally, delaying the onset of the tone embedded within the TINN stimulus can provide information about temporal, as well as spectral, processing. For these reasons, we have adopted the TINN stimulus in a novel approach to investigate neurophysiological mechanisms of auditory processing.

Previously, we have shown that systemic nicotine enhances the response to characteristic frequency (CF) stimuli, and reduces the response to a spectrally distant stimulus in rat and mouse primary auditory cortex (A1; Liang et al., 2008; Kawai et al., 2011; Intskirveli and Metherate, 2012). These results imply, but do not show directly, that nicotine narrows RFs in A1 and increases gain within the narrowed RF. Here, we used TINN-evoked responses to measure RF characteristics and directly show nicotine-induced increased gain within narrowed RFs. Moreover, modulation of RFs in A1 by systemic nicotine is the result of distinct nicotinic effects at several levels of the auditory pathway, including midbrain, thalamus, the thalamocortical pathway and cortex, that integrate to produce the overall effect observed in A1.

## Materials and Methods

### Animals

Adult (60- to 90-d-old) male FVB mice were used for all procedures in accordance with the National Institutes of Health Guide for the Care and Use of Laboratory Animals and as approved by the University of California, Irvine Institutional Animal Care and Use Committee. Mice were anesthetized with urethane (0.7 g/kg i.p.; Sigma) and xylazine (13 mg/kg i.p.; Phoenix Pharmaceutics), placed in a sound-attenuating chamber (AC-3; IAC), and maintained at 37°C. Anesthesia was supplemented as necessary with urethane (0.13 g/kg) and xylazine (1.3 mg/kg) via an intraperitoneal catheter to avoid movement of mice. Note that urethane anesthesia does not suppress nAChR function, unlike anesthetics such as barbiturates and ketamine (Hara and Harris, 2002; Tassonyi et al., 2002). The head was secured in a stereotaxic frame (model 923; Kopf Instruments) with blunt earbars. After a midline incision, the skull was cleared and secured to a custom head holder. A craniotomy was performed in the appropriate region for electrode placement or microinjections and the exposed brain was kept moist with warmed saline. After the craniotomy, the blunt earbars were removed to permit acoustic stimulation.

### Electrophysiology and acoustic stimulation

**Figure 1.**
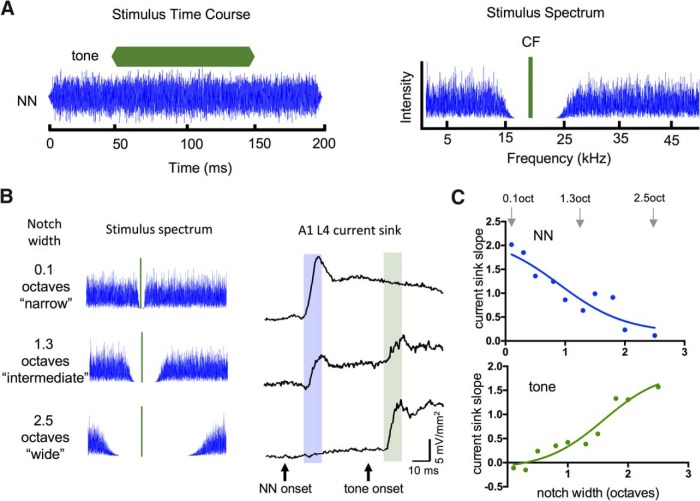
TINN stimulus used to estimate RF widths and suppression of tone-evoked responses. ***A***, Example TINN stimulus illustrates time course (left) and spectrum (right) of CF tone (green) and NN (blue). Spectral notch was varied in ∼0.25 octave steps (0.1-2.5 octaves) and centered (using octave scale) at CF. ***B***, Example TINN stimuli and evoked current sinks in L4 of A1; narrow-notch TINN evoked strong response to NN and no response to tone (top row), intermediate-notch TINN evoked weak response to NN and weak response to tone (middle row), wide-notch TINN evoked no response to NN and strong response to tone (bottom row). Shading indicates 10-ms analysis window for NN and tone responses. ***C***, Example analysis of TINN-evoked responses shows current-sink slope versus notch width for NN-evoked responses (top; arrows indicate data points from traces in ***B***) and tone-evoked responses (bottom), with data fitted by sigmoidal curves. Sigmoidal functions provide estimates of RF shape (top) and suppression of tone-evoked response by preceding RF stimulation (bottom). In this and the following figures, current sink slope is in units of mV mm^−2^ ms^−1^.

Stimulus-evoked local field potentials (LFPs) were recorded with a glass micropipette filled with 1 M NaCl (∼1 MΩ at 1 kHz) for locating auditory regions, or a 16-channel silicon multiprobe (∼2–3 MΩ at 1 kHz for each 177-μm^2^ recording site, 100-μm separation between recording sites; NeuroNexus Technologies). Recordings were filtered and amplified (1 Hz to 1 kHz, AI-401 or AI-405, CyberAmp 380; Molecular Devices), digitized (5 kHz), and stored on a computer (Apple Macintosh running AxoGraph software). Acoustic stimuli were digitally synthesized and controlled with custom MATLAB software (Dr Tom Lu, Center for Hearing Research Computing and Engineering Core) and delivered through an open-field speaker (ES-1 or FF-1 with ED-1 driver; Tucker-Davis Technologies) positioned ∼3 cm in front of the left ear. For calibration [sound pressure level (SPL), in dB re: 20 μPa] a microphone (model 4939 and Nexus amplifier; Brüel and Kjaer) was positioned in place of the animal at the tip of the left earbar. TINN stimuli (Fig. 1*A*) consisted of a tone at the CF of the recording site, embedded within notched noise (notch centered at CF). CF tones were 100 ms duration (5 ms linear rise and fall ramps), frequency range 10-20 kHz, 15 dB above threshold and onset 50 ms after NN onset. NN component was 200 ms in duration with 5 ms linear rise and fall ramps, variable notch size (0.1-2.5 octaves), fixed amplitude (set at 25 dB above threshold for white noise) and overall frequency range 1-50 kHz. For data collection, TINN stimuli were delivered at a rate of 0.5/s in sets of 25 trials.

### Determination of recording sites

A1. The craniotomy was centered ∼3 mm posterior, 4 mm lateral, and 2 mm ventral to bregma. To identify A1, we recorded tone-evoked responses from multiple sites ∼250 μm apart along the anterior-posterior (AP) axis in auditory cortex, using a glass micropipette inserted into layer 4 (L4; ∼400-μm depth) and orthogonal to the cortical surface. Based on responses to a standard set of tones (1-40 kHz in 2.5 kHz steps, −10 dB to 70 dB SPL in 5 dB steps), we determined CF (frequency with the lowest threshold) for each recording site. CF maps were constructed to identify the tonotopy expected for A1, including a reversal of tonotopy at the border with the anterior auditory field (Stiebler et al., 1997). We then chose a site within A1 having a CF of 10–20 kHz (so that TINN stimuli could be constructed with the spectral notch centered near the middle (in octaves) of our frequency range of 1-50 kHz), and mapped along the dorsal-ventral (DV) axis of the presumed isofrequency region to find the site with the shortest-latency, largest-amplitude surface LFP (i.e., isofrequency region mapped using a micropipette placed on the cortical surface). This site was used for all subsequent procedures. We inserted the 16-channel multiprobe perpendicular to the cortical surface to record LFPs throughout the cortical depth, and re-determined CF more precisely (steps of 1 kHz and 5 dB) based on the initial slope and onset latency of LFPs recorded 300–400 μm below the surface. Tone-evoked LFPs were considered threshold responses when their amplitude exceeded 3 standard deviations of the mean baseline (determined over the 100 ms preceding the tone).

Medial geniculate, ventral division (MGv). We mapped the MG body with a glass micropipette angled 20 degrees from horizontal and inserted through auditory cortex, starting ∼3 mm posterior to bregma and mapping in the AP, DV, and medial-lateral (ML) planes until the expected tonotopy for the MGv was identified (Hackett et al., 2011). We then inserted a 16-channel multiprobe at the same angle so that several channels would span MGv, selected a channel with clear tone-evoked responses, and re-determined CF using similar methods as for A1.

Inferior colliculus, central nucleus (ICc). A glass micropipette was inserted vertically ∼1 mm lateral and ∼1 mm posterior to lambda, and we mapped along AP, DV, and ML axes to identify the tonotopy expected for the ICc (Stiebler and Ehret, 1985). We then inserted a 16-channel multiprobe vertically so that multiple channels would span the IC, selected a channel with clear tone-evoked responses, and re-determined CF, as above.

To confirm placement of the multiprobe in MGv or ICc, after each experiment the animal was perfused with 4% paraformaldehyde, the brain was removed and sectioned in the “thalamocortical” (MGv; Cruikshank and Rose, 2002) or transverse (ICc) plane, and the multiprobe track visualized and confirmed to pass through the appropriate structure.

### Drug administration

For systemic injections, nicotine ditartrate (Tocris) was dissolved in saline (2.1 mg/kg free base), and delivered subcutaneously. Since the effects of systemic nicotine on tone-evoked responses in A1 last 30 min or longer (Kawai et al., 2011; Intskirveli and Metherate, 2012), all postnicotine data were obtained within 20 min. For intracerebral microinjections, nicotine was dissolved in artificial cerebrospinal fluid (ACSF; 125 mM NaCl, 2.5 mM KCl, 25 mM NaHCO_3_, 1.25 mM KH_2_PO_4_, 1.2 mM MgSO_4_, 2.0 mM CaCl_2_, and 10 mM dextrose) to a final concentration of 10 μM. Similarly, NS9283 (3-[3-(3-pyridinyl)-1,2,4-oxadiazol-5-yl]benzonitrile, Tocris) was dissolved in dimethyl sulfoxide (DMSO) for a stock concentration of 10 mM, with a final dilution in ACSF to 10 μM (0.1% DMSO). Vehicle control injections were performed with either ACSF or 0.1% DMSO. All microinjection solutions also contained 2% tetramethylrhodamine dextran (10 kDa, Invitrogen) or fluorescein dextran (10 kDa, Invitrogen) to mark injection sites. Muscimol (5-aminomethyl-3- 155 hydroxyisoxazole, Sigma) was dissolved in ACSF (100-200 μM, 1 µl) and applied to the cortical surface near the entry point of the multiprobe using a 1 µl Hamilton syringe. For intracerebral injections, we used a 0.5 µl Hamilton syringe fitted with a micropipette (∼20 µm tip). Intracortical injections were within 100 μm of the multiprobe, and injections in the superior thalamic radiation (STR) targeted a location 1.6 mm posterior, 2.3 mm lateral, and 2.8 mm ventral to bregma. To confirm injection sites, after experiments and animal perfusion the brain was removed and sectioned in the thalamocortical (STR injections) or coronal (cortical injections) plane. The brightest (center) region of fluorescence was designated the injection site.

### Data analysis

Stimulus-evoked responses were the average of 25 trials. Current-source density (CSD) profiles were constructed off-line as described previously (Intskirveli and Metherate 2012). One dimensional CSD profiles are the second spatial derivative of the LFP laminar profile (Müller-Preuss and Mitzdorf, 1984); conventionally, a current sink implies the location, timing and magnitude of underlying synaptic excitation. The response onset was defined as the time at which the CSD trace crossed a threshold 2x SD above baseline. The middle-layer current sink with shortest onset latency was designated L4, and selected for subsequent analysis. For both CSD and LFP traces, the initial slope was measured over the 10 ms following response onset (50 data points). The L4 current sink reflects monosynaptic thalamocortical input as well as intracortical activity, as demonstrated recently using a titrated dose of the GABA agonist muscimol to suppress intracortical activity but not monosynaptic inputs (Intskirveli et al., 2016); as a result, our 10-ms analysis window includes both response types. Slope data were analyzed and plotted using GraphPad Prism, with slopes normalized to the plateau value of a sigmoidal curve fit to the data from each animal. Group RF data were compared using repeated-measures two-way ANOVA (α = 0.05) and sorted into bins of 0.3 octaves for plotting. Mean values are presented ±SEM, and “n” values represent number of mice. Multiunit activity (MUA) was estimated by high-pass filtering LFP data at 500 Hz, rectifying and averaging responses across 25 trials, and smoothing the result using a Gaussian filter width of 5 ms.

## Results

### TINN-evoked responses in A1

TINN stimuli traditionally are used in psychoacoustics to estimate perceptual filters (Patterson, 1976), but are used here to provide an electrophysiological measure of RF structure and dynamics (Fig. 1*A*). Our TINN stimulus has two components: a tone set to the CF of the recording site (15 dB above CF threshold), and a NN component with the spectral notch centered on CF (Fig. 1*A*, right; noise range 1-50 kHz, fixed amplitude, notch range 0.1-2.5 octaves). Tone and NN onsets are asynchronous, with the tone beginning 50 ms after the NN (Fig. 1*A*, left). This arrangement provides two advantages over simultaneous onset: first, the NN evokes a response that precedes tone onset (Fig. 1*B*, right), and therefore can be attributed solely to the NN stimulus; and second, the 50 ms delay allows for development of NN-evoked inhibition, potentially including both feedforward and lateral inhibition (Semple, 1995; Sutter et al., 1999; Wehr and Zador, 2003, 2005). Thus, use of the TINN stimulus allowed us to simultaneously assess important spectral and temporal characteristics of the RF.

We inserted a 16-channel linear multiprobe into A1 to record stimulus-evoked LFPs throughout the cortical depth at 100 µm intervals, and subsequently derived CSD profiles offline, as previously described (Kawai et al., 2011; Intskirveli and Metherate, 2012). For this study, we identified and focused on the shortest-latency current sink in the middle layers, which we refer to as the L4 current sink. This current sink reflects monosynaptic thalamocortical input from the medial geniculate body as well as subsequent intracortical activity, and our 10-ms analysis window includes both response types (Materials and Methods). We quantified TINN-evoked responses by measuring the slope of the L4 current sink over the first 10 ms after response onset, separately for the NN component (Fig. 1*B*, right, blue shaded area in example traces), and for the CF tone (Fig. 1*B*, green shaded area). We then plotted response slope versus notch width, separately for each component (Fig. 1*C*).

The function obtained by plotting NN-evoked responses versus notch width (Fig. 1*C*, top) provides an estimate of the RF for a recording site, analogous to that obtained using a sequence of pure tones but with the advantage that NN stimuli activate multiple frequency channels simultaneously, i.e., a more naturalistic stimulus. Then, the response to the CF tone provides information about RF dynamics (Fig. 1*C*, bottom). For example, at narrow notch widths, the strong NN-evoked response is followed by little or no response to the tone (Fig. 1*B*, top row), presumably because both the NN and tone stimuli activate largely overlapping frequency channels. At intermediate notch widths, the NN-evoked response is reduced and the tone-evoked response begins to emerge (Fig. 1*B*, middle row). At wide notch widths, the NN-evoked-response is weak or nonexistent and exerts little effect on the tone-evoked response (Fig. 1*B*, bottom row). Plotting the magnitude of the tone-evoked response versus notch width (Fig. 1*C*, bottom) provides a quantitative estimate of suppression by the NN stimulus, which complements the RF measure (Fig. 1*C*, top).

### Nicotinic modulation of TINN-evoked responses

**Figure 2.**
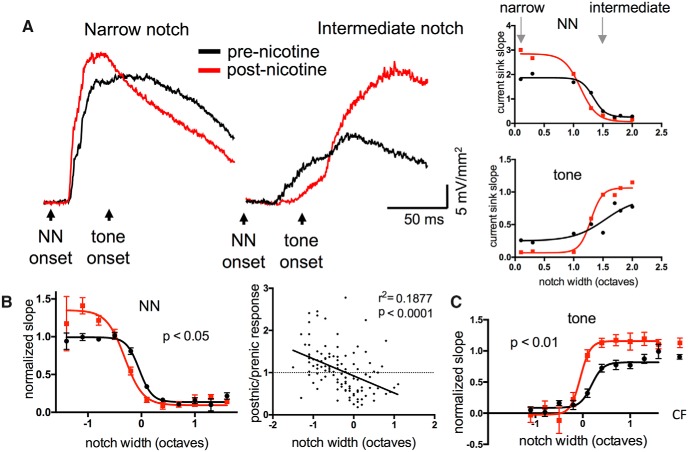
Systemic nicotine enhanced responses to narrow-notch stimuli and tone stimuli, and reduced responses to intermediate-notch stimuli in A1. ***A***, Example of nicotine effect on TINN-evoked current sinks in L4 (left) and derived functions (right). ***B***, Group data (*n* = 23) demonstrating nicotinic enhancement of responses to narrow-notch stimuli and reduction of responses to intermediate-notch stimuli (left). Data are normalized to prenicotine plateau of sigmoid function. Group data are also plotted as postnicotine/prenicotine response ratios versus notch width (right), indicating differential nicotinic effects at narrow versus intermediate notch sizes. ***C***, Group data (*n* = 18) demonstrating nicotinic enhancement of responses to CF tone contained within TINN stimulus (sigmoidal curves) and to CF tone presented alone (separated data points on right).

We recorded TINN-evoked current sinks in L4 before and after systemic administration of nicotine (2.1 mg/kg, s.c.), with postnicotine responses obtained within 20 min, i.e., before nicotine effects dissipated (Kawai et al., 2011). We analyzed NN-evoked responses (before tone onset), fitting the data with a sigmoid function, and found a drug effect that varied with notch width: nicotine enhanced responses to narrow-notch stimuli and reduced responses to intermediate-notch stimuli (Fig. 2*A*, group data for 23 animals in Figure 2*B*, left). To obtain the average RF across animals (Fig. 2*B*), individual sigmoid functions were aligned using the notch width corresponding to the half-maximal, prenicotine response (Fig. 2*A*, notch width of ∼1.3 octaves); this reference notch width is plotted as “0 octaves” in Figure 2*B*. A repeated-measures two-way ANOVA showed a main effect of notch width (*n* = 23, *F*_(30,152)_ = 35.66, *p* < 0.0001), a main effect of nicotine (*F*_(1,152)_ = 5.288, *p* = 0.023), and an interaction term reflecting different effects of nicotine at narrow versus intermediate notches (*F*_(30,152)_ = 2.646, *p* < 0.0001). In contrast, control injections of saline had no effect (data not shown; *n* = 11, saline main effect *F*_(1,53)_ = 1.834, *p* = 0.18). Nicotine’s opposite effects for narrow-notch versus intermediate-notch stimuli shifted the sigmoidal RF function to narrower widths and higher slope plateau values, indicating increased gain within a narrowed RF [Fig. 2*B*; sigmoid function fitted to mean data shifted 0.27 octaves to left (at 50% max) and to 36% higher slope plateau value].

In keeping with the descriptions above, we will refer to notch widths as “narrow” (eliciting the maximal plateau response), “intermediate” (near the reference notch width), and “wide” (eliciting no response). Thus, NN stimuli with intermediate notch widths stimulate only the RF edges, and NN stimuli with wide notch widths stimulate outside the RF (evidenced by no effect on tone-evoked responses, which reach their maximal plateau level; Fig. 2*C*).

A related analysis of nicotine’s effects, shown in Figure 2*B*, right, presents NN-evoked responses as postnicotine/prenicotine ratios. These are the same data used for average RFs (Fig. 2*B*, left), but with individual pre- and postnicotine data expressed as a ratio (to avoid meaningless ratios, only responses with predrug values >2 SD above noise levels are included). Consistent with the results shown for average RFs (Fig. 2*B*, left), this analysis reveals a tendency for normalized slope responses to narrow-notch stimuli to be enhanced (ratio >1) and responses to intermediate-notch stimuli to be reduced (ratio <1; *r* ^2^ = 0.1850, *p* < 0.0001).

We next analyzed NN-evoked suppression of responses to CF tones, and found that nicotine reduced suppression of, and/or overtly enhanced, tone-evoked responses (Fig. 2*A*,*C*). Nicotine did not change the near-complete suppression of tone-evoked responses for narrow-notch stimuli, but enhanced tone-evoked responses for intermediate- and wide-notch stimuli (Fig. 2*C*; repeated-measures two-way ANOVA, *n* = 18, nicotine main effect *F*_(1,113)_ = 7.954, *p* < 0.01, interaction *F*_(30,113)_ = 1.210, *p* < 0.05). Again, saline injections had no effect (not shown, *n* = 10, *F*_(1,45)_ = 1.953, *p* = 0.17). Figure 2*C* also shows the response to the CF tone presented by itself (without NN stimulation), demonstrating that the “plateau” response to the tone presented within NN is unaffected by wide-notch stimuli; nicotine enhanced responses to CF tone alone, indicating that nicotinic effects on tone for wide-notch stimuli result from an overt enhancement rather than reduced suppression (Fig. 2*C*, right; paired *t* test, *n* = 23, pre-nic mean = 0.93 ± 0.04, post-nic mean = 1.18 ± 0.08, *t*_18_ = 2.893 *p* = 0.01). The overall results from TINN-evoked responses indicate that nicotine increased gain within a narrowed RF (Fig. 2*B*), which in turn reduced suppression of CF-evoked responses by intermediate-notch stimuli, and enhanced CF-evoked responses following wide-notch stimuli (Fig. 2*C*).

**Figure 3.**
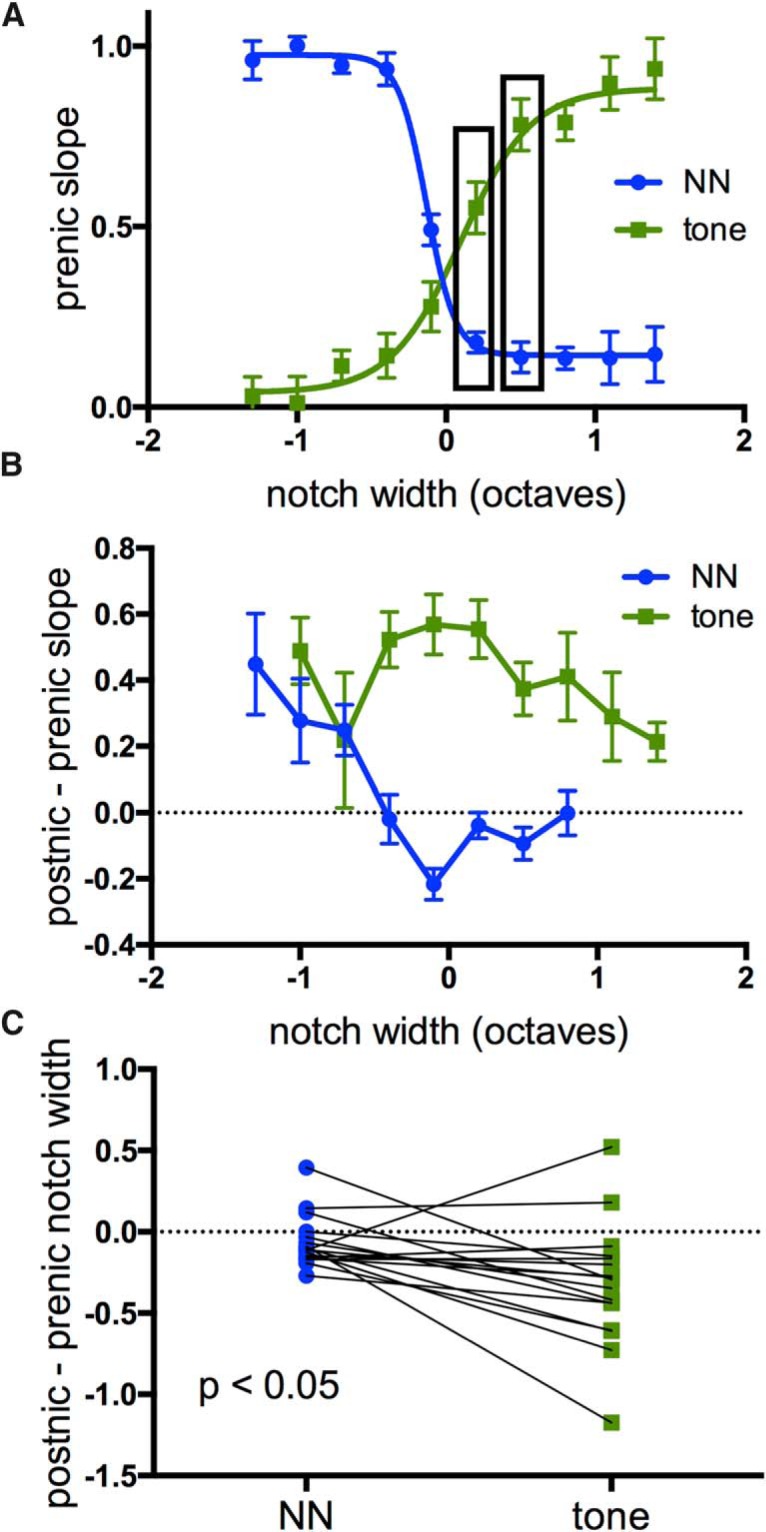
A closer look at responses to intermediate-notch TINN stimuli. ***A***, Superimposed group data for prenicotine responses emphasize substantial suppression of tone-evoked responses, even by relatively weak NN-evoked responses (boxed data points). ***B***, Effects of nicotine on normalized response values (postnicotine minus prenicotine slope) showing reduction of NN-evoked responses at intermediate notch widths and enhancement of tone-evoked responses over a wider range of notch sizes. ***C***, Nicotine-induced shift in width of sigmoid functions (in octaves, measured at 50% max), i.e., RF and tone-suppression functions, reveal larger shift in tone-suppression function.

Because nicotine’s effects at intermediate notch widths were complex (reduced response to NN stimulus, enhanced response to tone), we examined this more closely. Figure 3*A* superimposes prenicotine functions for NN- and tone-evoked responses (same functions as in Figure 2*B*,*C*). The graph shows that NN stimulation of the RF edges produced relatively weak responses, as might be expected, yet substantial reduction of the tone-evoked response 50 ms later (Fig. 3*A*, data highlighted by boxes). The net change produced by nicotine is shown in Figure 3*B*, separately for NN-evoked responses (blue data points) and tone-evoked responses (green; same data as in Figure 2*B*,*C* but expressed as difference functions). Reduction of the NN-evoked response is greatest near the RF edge, whereas enhancement of the tone-evoked response occurs over a wider range of notch widths, including stimulation outside the RF. Thus, the altered tone-evoked response likely results from overt enhancement (increased gain), as well as reduced suppression due to a narrowed RF and, possibly, altered lateral inhibition (Discussion).

The nicotine-induced shift in RF width (Fig. 2*B*) and tone-suppression function (Fig. 2*C*) for each animal are quantified in Figure 3*C* (measured at half-maximal values). Nicotine produced a greater shift of the tone-suppression function, suggesting that the change in tone-evoked response is not fully accounted for by RF narrowing (RF shift 0.06 ± 0.04 octaves, tone-suppression shift 0.32 ± 0.09 octaves, paired *t* test, *n* = 18, *t*_16_ = 2.818, *p* = 0.012).

**Figure 4.**
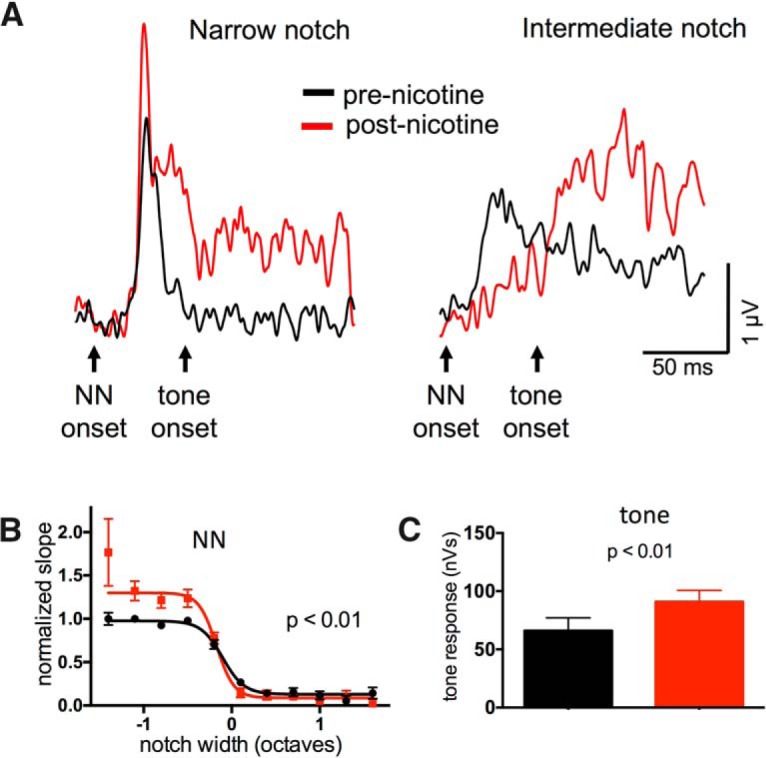
Systemic nicotine produced similar effects on TINN-evoked MUA. ***A***, Example of nicotine effect on TINN-evoked MUA in L4 of A1. ***B***, Group data (*n* = 21) demonstrating nicotinic enhancement of MUA evoked by narrow-notch stimuli and reduction of MUA responses to intermediate-notch stimuli. ***C***, Group data (*n* = 18) of tone-evoked MUA responses (wide-notch TINN stimuli), illustrating nicotinic enhancement.

We also examined the effect of nicotine on TINN-evoked MUA, since the L4 current sink largely reflects synaptic activity (neural input), whereas MUA reflects neural output. MUA was estimated by high-pass filtering (>500 Hz) and rectifying the evoked LFP response in L4, and then integrating the resulting trace over the 50 ms following either NN or tone onset. As with the L4 current sink, nicotine enhanced NN-evoked MUA for narrow-notch stimuli and reduced MUA for intermediate-notch stimuli (Fig. 4*A*,*B*; *n* = 21, repeated-measures two-way ANOVA, main notch effect *F*_(30,130)_ = 25.14, *p* < 0.0001, main nicotine effect *F*_(1,130)_ = 8.427, *p* = 0.004, interaction *F*_(30,130)_ = 2.764, *p* < 0.0001). Nicotine also appeared to enhance tone-evoked MUA for intermediate-notch stimuli (Fig. 4*A*, right), however this effect was more difficult to discern for MUA activity integrated over 50 ms than for current-sink initial slopes (measured over 10 ms). We therefore measured, for each animal, tone-evoked MUA at the notch width associated with the greatest enhancement of current-sink slope, and found MUA enhancement as well (Fig. 4*C*, paired *t* test, *t*_15_ = 3.495, *p* = 0.003). These results show that nicotinic regulation of TINN-evoked responses is similar for both CSD and MUA measures.

### Nicotine modulation of TINN-evoked responses in subcortical regions

Since nicotine was delivered systemically, its effects recorded in A1 could originate outside the cortex. Thus, we sought to determine if effects in A1 were inherited from subcortical regions. We applied the GABA-A receptor agonist muscimol (100-200 μM) to the cortical surface and recorded L4 current sinks evoked by TINN stimuli. This dose of muscimol was recently shown to be optimal for silencing intracortical activity while preserving L4 responses to monosynaptic thalamocortical input (Intskirveli et al., 2016). Our rationale was that if effects recorded in A1 depend on nicotinic actions on intracortical circuits, then applying muscimol before nicotine would preclude those effects.

**Figure 5.**
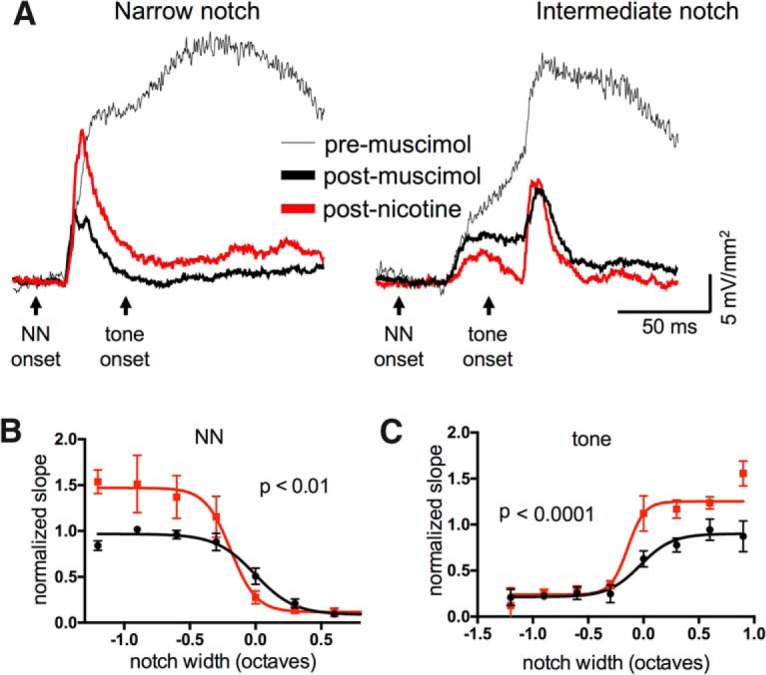
Systemic nicotine effects occurred despite the presence of intracortical muscimol. ***A***, Example of nicotine effect on TINN-evoked L4 current sink in the presence of muscimol (100 µM) to silence intracortical activity and isolate presumed thalamocortical input. ***B***, Group data (*n* = 8) demonstrating that nicotinic effects on NN-evoked responses occurred in the presence of muscimol. ***C***, Nicotinic enhancement of tone-evoked responses also occurred after muscimol (*n* = 8).

Muscimol did not affect the initial slope of NN-evoked current sinks, but strongly reduced longer-latency components (Fig. 5*A*, paired *t* test, *n* = 8; initial slope *t*_7_ = 1.665, *p* = 0.139; amplitude at 100 ms *t*_7_ = 2.304, *p* = 0.0052), consistent with our expectation that muscimol can suppress intracortical activity without affecting thalamocortical inputs (Intskirveli et al., 2016). However, the subsequent administration of systemic nicotine produced changes to the NN-evoked responses similar to those observed in the absence of muscimol (Fig. 5*A*,*B*; *n* = 8, repeated-measures two-way ANOVA, main notch effect *F*_(22,35)_ = 9.379, *p* < 0.0001, main nicotine effect *F*_(1,35)_ = 8.751, *p* = 0.006, interaction effect *F*_(22,35)_ = 1.446, *p* = 0.16). Similarly, nicotine enhanced tone-evoked responses following intermediate- and wide-notch stimuli (Fig. 5*C*; main nicotine effect *F*_(1,34)_ = 28.43, *p* < 0.0001, interaction effect *F*_(23,34_= 2.661, *p* = 0.005). Thus, the qualitatively similar effects indicated that some nicotinic effects do not require intracortical activity.

**Figure 6.**
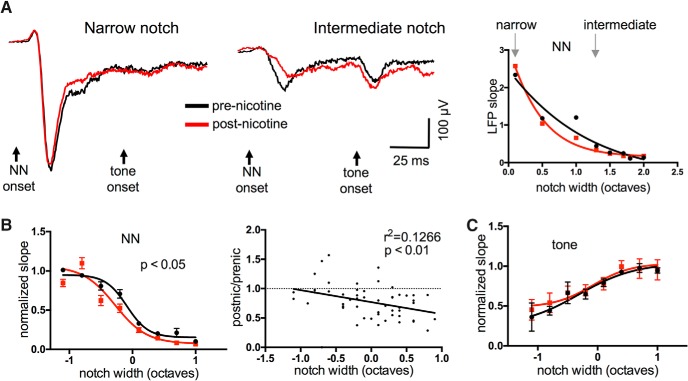
In ICc, systemic nicotine reduced responses to intermediate-notch NN stimuli but did not enhance any TINN-evoked response. ***A***, Example of nicotine effect on TINN-evoked LFPs in ICc. Units of LFP slope are µV/ms. ***B***, Group data (*n* = 8) demonstrating nicotinic reduction of responses to NN stimuli (left). Group data are also plotted as postnicotine/prenicotine response ratios (right), illustrating that the reduction occurs primarily at wider notches. ***C***, Group data (*n* = 8) showing no effect on tone-evoked responses.

To identify potential subcortical loci of nicotinic actions, we recorded from the central nucleus of the inferior colliculus (ICc). Following an initial microelectrode mapping to identify the ICc based on the expected tonotopic progression of CFs (Stiebler and Ehret, 1985), we inserted a linear multiprobe into the ICc from the dorsal surface and selected a recording site with a clear CF and strong evoked responses (Fig. 6*A*). ICc location was confirmed with *post hoc* histology to reconstruct the multiprobe track. We analyzed LFP recordings, since the assumptions for one-dimensional CSD analysis may not hold true for subcortical structures, but still measured response slope over the first 10 ms after response onset. For NN-evoked responses, systemic nicotine had little effect on the response to narrow-notch stimuli, but reduced the response to intermediate-notch stimuli; notably, nicotine did not produce clear enhancement of any NN-evoked response (Fig. 6*A*,*B*; repeated-measures two-way ANOVA, *n* = 8, main notch effect *F*_(22,38)_ = 40.67, *p* < 0.0001, main nicotine effect *F*_(1,38)_ = 5.970, *p* = 0.019, interaction *p* = 0.12). Examination of postnicotine/prenicotine response ratios confirmed that nicotine’s suppressive effect varied with notch width (Fig. 6*B*, right; *r* ^2^ = 0.1266, *p* = 0.009). However, nicotine had no effect on tone-evoked responses (Fig. 6*A*,*C*; main nicotine effect *p* = 0.46). Finally, saline injections produced no effect on any TINN-evoked response (not shown, *n* = 3, *F*_(1,10)_ = 3.723, *p* = 0.09). Thus, the effects of systemic nicotinic in ICc were largely suppressive, varied with notch width, and, notably, did not facilitate any TINN-evoked response component.

**Figure 7.**
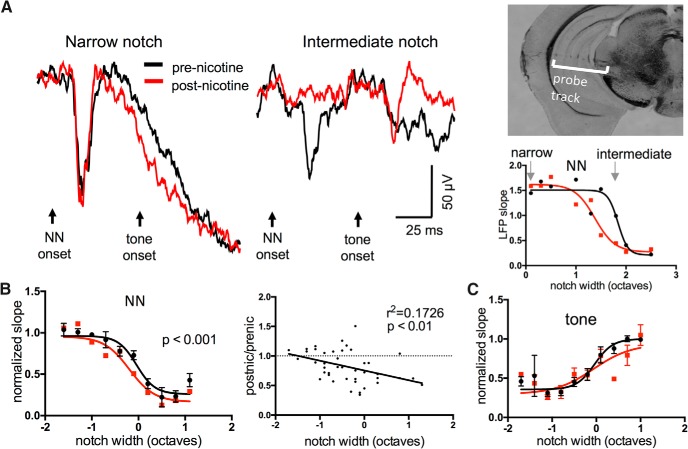
In MGv, systemic nicotine reduced responses to intermediate-notch NN stimuli but did not enhance any TINN-evoked response. ***A***, Example of nicotine effect on TINN-evoked LFPs in MGv (left, middle), coronal brain slice showing recording probe track (inset), and sigmoidal NN functions (right; units of LFP slope are µV/ms.). ***B***, Group data (*n* = 7) demonstrating nicotinic reduction of responses to NN stimuli (left). Group data are also plotted as postnicotine/prenicotine response ratios (right), illustrating that the reduction occurs primarily at wider notches. ***C***, Group data (*n* = 7) showing that nicotine has no effect on tone-evoked responses.

Since ICc recordings revealed no evidence for nicotinic facilitation or altered tone-evoked responses, we next recorded “downstream” to the ICc in the ventral division of the medial geniculate body (MGv). As with IC experiments, following microelectrode mapping we inserted a multiprobe from the lateral surface of the brain into the MG to record LFPs, and confirmed recording sites based on the expected progression of CFs (Hackett et al., 2011) and *post hoc* visualization of the multiprobe track (Fig. 7*A*). The effects of systemic nicotine were similar to those seen in the IC: nicotine had little effect on narrow-notch NN-evoked responses, reduced responses to intermediate-notch stimuli, and had no effect on tone-evoked responses (Fig. 7*A-C*; NN-evoked responses: *n* = 7, repeated-measures two-way ANOVA, main notch effect *F*_(24,30)_ = 13.83, *p* < 0.0001, main nicotine effect *F*_(1,30)_ = 14.29, *p* = 0.0007, interaction effect *p* = 0.7; tone-evoked responses: main nicotine effect *p* = 0.32). The postnicotine/prenicotine response ratio again showed that the drug effect increased with notch width (Fig. 7*B*, right; *n* = 7, *r* ^2^ = 0.1726, *p* = 0.006). Saline controls showed no effect (not shown, *n* = 5, main saline effect *F*_(1,29_ = 0.4884, *p* = 0.30). The effects of systemic nicotine in MGv therefore resembled those seen in ICc in that they were primarily suppressive, varied with notch width, and did not facilitate any response component.

**Figure 8.**
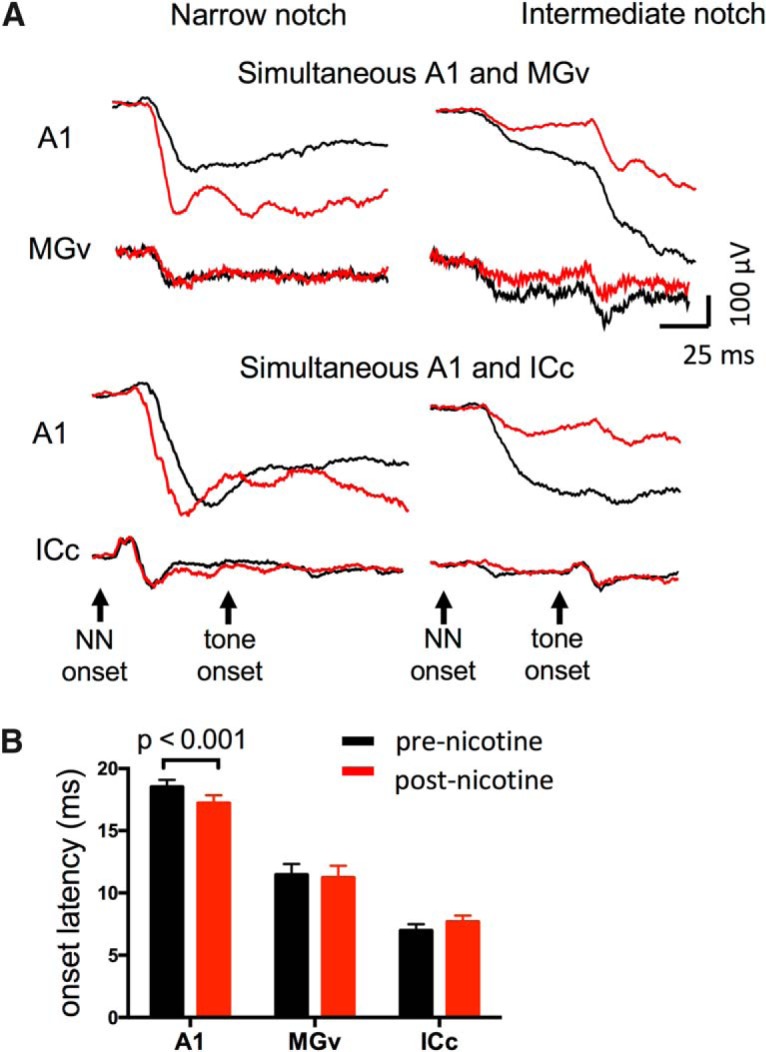
Simultaneous recordings in A1 and ICc or MGv confirm differential effects of systemic nicotine. ***A***, Examples of nicotine effects on TINN-evoked LFPs in A1 and MGv (top), and in A1 and ICc (bottom), confirming that nicotinic enhancement of TINN-evoked responses occurred only in A1, whereas nicotinic reduction of responses occurred in all three regions. ***B***, Onset latencies in A1, MGv, and ICc (0.1 octave NN stimulus). Systemic nicotine reduced onset latency only in A1.

The apparent differential effects of systemic nicotine in A1 versus subcortical regions, i.e., only in A1 was there enhancement of responses to narrow-notch stimuli and CF tones, was reinforced in 5 animals with simultaneous recordings in A1 and either MGv (*n* = 2; Fig. 8*A*, top) or ICc (*n* = 3; Fig. 8*A*, bottom). For this direct comparison, we examined only LFPs in each region (rather than converting to CSDs in A1). In each case, nicotine enhanced the response to narrow-notch NN stimuli in A1, but not in subcortical regions. For intermediate-notch stimuli, nicotinic reduction of responses was seen at all recording sites. These simultaneous recordings reinforce the conclusion that nicotinic facilitation occurs downstream to processing in ICc and MGv.

An additional comparison across auditory regions concerned the onset latency of NN-evoked responses. Prior studies have noted nicotinic reduction of onset latency in A1 for tone-evoked responses (Liang et al., 2006; Kawai et al., 2011; Intskirveli and Metherate, 2012) and isolation of the thalamocortical pathway *in vitro* demonstrated nicotinic reduction of spike latency for MG-evoked axon spikes (Kawai et al., 2007). In the present study, onset latency was determined in each region for the narrowest-notch (0.1 octave) NN-evoked response. Systemic nicotine reduced onset latency in A1 (Fig. 8*B*, *n* = 23, paired *t* test, *t*_22_ = 3.977, *p* = 0.0006), but had no effect in MGv or ICc (*n* = 7, *t*_6_ = 0.7035, *p* = 0.51, and *n* = 8, *t*_7_ = 1.543, *p* = 0.16, respectively). Again, these results are consistent with the notion that nicotinic facilitation occurs downstream to processing in ICc and MGv.

Overall, the data indicate that the subcortical effects of systemic nicotine are largely suppressive and act to narrow RFs in auditory relay nuclei. We conclude that at least a portion of nicotinic narrowing of RFs in A1 is inherited from subcortical regions. However, we did not observe nicotinic modulation of tone-evoked responses in subcortical regions, nor facilitation of any TINN-evoked response; these issues will be addressed further, below, after a comparison of response characteristics across auditory regions.

### Comparison of RFs and TINN-evoked response features across A1, MGv, and ICc

**Figure 9.**
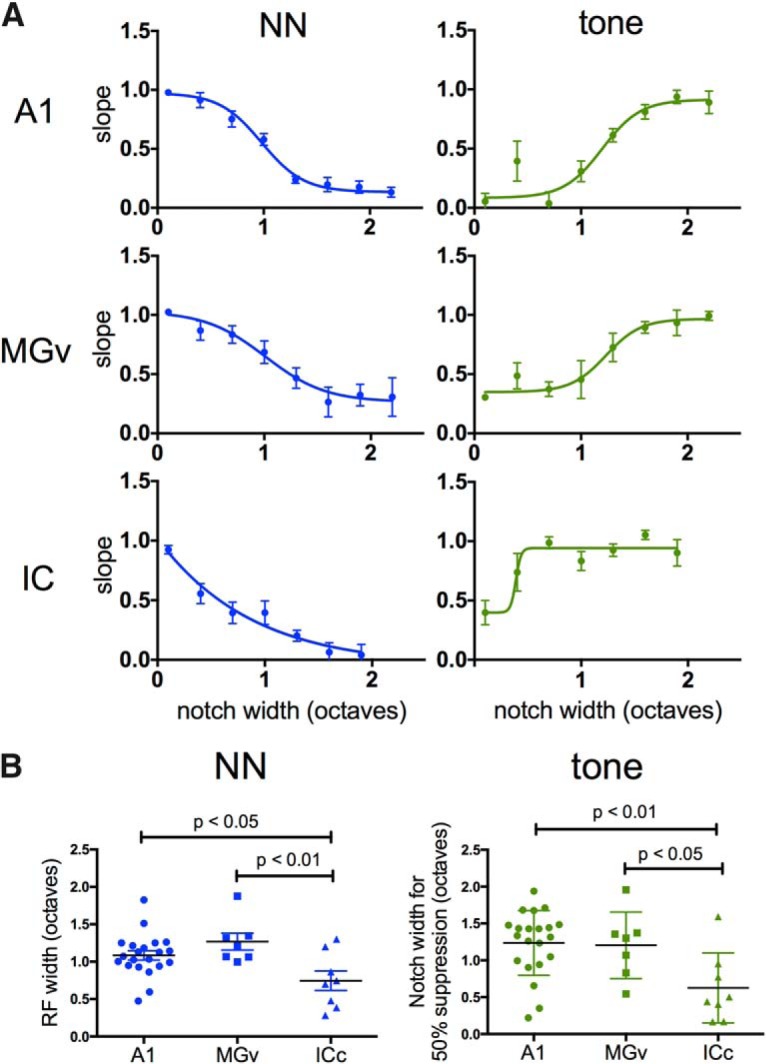
Comparison of RFs and response suppression in A1, MGv, and ICc. ***A***, NN-evoked (left) and tone-evoked (right) LFP response functions in A1, MGv, and ICc. Response magnitude normalized to plateau value for each animal; notch width is absolute value (unlike previous figures). ***B***, Comparison of RF width and suppression of tone-evoked response, based on notch width producing 50% max response in A; individual data points are shown with group means. ICc exhibited narrower RFs and suppression of tone-evoked responses at narrower notch widths.

The use of similar techniques to record TINN-evoked responses in A1, MGv, and ICc provides an opportunity to compare response features across the three regions (as for onset latency, above). We therefore compared RF functions derived from NN-evoked responses, as well as NN-evoked suppression of tone-evoked responses. The results in Figure 9 consist of the same data represented in previous figures but with two differences to facilitate comparison: first, response magnitudes are plotted as a function of absolute notch width (rather than aligned to a reference notch width), and second, A1 data are derived from LFP recordings, rather than current sinks (note that RF widths derived from LFPs (Fig. 9) do not differ from RF widths derived from CSDs for the same recording sites (Fig. 2*B*); paired *t* test, *n* = 10, *p* = 0.68).

To compare RF width across the three regions, for each animal we determined the notch width that produced the half-maximal, NN-evoked response (i.e., the reference notch width; Fig. 2*B*). Individual RF widths are plotted in Figure 9*B*, left, grouped by region. RF widths in A1 and MGv were similar, but wider than in ICc (one-way ANOVA with Tukey’s multiple comparison test: A1 versus MGv, *p* = 0.36; A1 versus ICc, *p* = 0.03; MGv versus ICc *p* = 0.006). We used a similar approach to compare NN-evoked suppression of tone-evoked responses, and determined the notch width for each animal that produced 50% suppression (Fig. 9*A*, right, half-maximal response for the sigmoid functions). The results are in Figure 9*B*, right. Again, the notch width was similar in A1 and MGv, and narrower in ICc (one-way ANOVA with Tukey’s multiple comparison test: A1 versus MGv, *p* = 0.98; A1 versus ICc, *p* = 0.007; MGv versus ICc, *p* = 0.046). Overall, these results show that RF widths in A1 and MGv are similar, averaging just over one octave, and wider than in ICc.

### Origin of nicotinic enhancement of TINN-evoked responses

**Figure 10.**
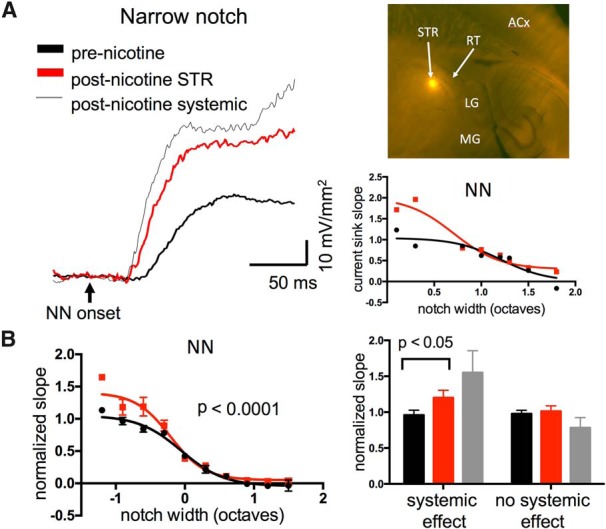
Nicotine microinjection in STR enhanced TINN-evoked responses in A1. ***A***, Example of NN-evoked current sink enhanced by nicotine microinjection in STR, and subsequently by systemic nicotine (left); graph (right) shows response magnitudes before and after STR microinjection, and inset shows *post hoc* visualization of fluorescent injection site in STR (horizontal plane; LG, lateral geniculate; RT, reticular nucleus; ACx, auditory cortex). ***B***, Group data (*n* = 10) showing enhancing effect of nicotine STR microinjections on NN-evoked responses (left). In a subset of six animals that appeared more sensitive to systemic nicotine, STR microinjections were effective, whereas in four animals that were insensitive to systemic nicotine STR microinjections also had no effect (right).

The results thus far show that systemic nicotine can reduce NN-evoked responses in subcortical auditory regions, yet the origin of nicotinic enhancement remains unclear. Nicotinic enhancement in A1 persisted in the presence of muscimol-induced silencing of intracortical circuits, yet was not observed in MGv (or ICc). Since a previous *in vitro* study found that nicotine increased the excitability of thalamocortical axons, but did not affect transmitter release at thalamocortical terminals (Kawai et al., 2007), we tested the involvement of the thalamocortical pathway in facilitating responses. To do so, we recorded L4 current sinks in A1 before and after microinjecting nicotine into the STR, a distinct white matter tract within the thalamus through which myelinated axons from MGv course on their way to A1 (Fig. 10*A*, inset). Nicotine microinjections were delivered using a micropipette attached to a Hamilton syringe, with each injection site visualized using a fluorescent dye (Fig. 10*A*); only data from injection sites centered within STR were considered further.

Microinjection of nicotine (10 µM, 50-100 µl) in STR enhanced A1 responses to narrow-notch NN stimuli, with little effect on responses to wider-notch stimuli (Fig. 10*A*,*B*; *n* = 10, repeated-measures two-way ANOVA, main notch effect *F*_(26,55)_ = 32.25, *p* < 0.0001, main nicotine effect *F*_(1,55)_ = 19.37, *p* < 0.0001, interaction effect *F*_(26,55)_ = 1.51, *p* = 0.10). Nicotine in STR also enhanced tone-evoked responses, similar to systemic effects recorded in A1 (not shown, repeated measures two-way ANOVA, *n* = 10, *F*_(1,41)_ = 4.593, *p* = 0.038) These data indicate that nicotinic actions in the thalamocortical pathway can enhance acoustic-evoked responses in A1, and explain, at least partly, how systemic nicotine can enhance responses in A1, but not MGv.

Although, on average, nicotine injected into STR enhanced the cortical response to narrow-notch stimuli, there was substantial individual variability. We minimized the impact of misplaced injections by including only data from injection sites centered in STR, but other factors likely played a role, notably the variable density of nAChRs within the thalamocortical pathway (Bieszczad et al., 2012). To control for inter-animal variability in sensitivity to nicotine, we followed each STR microinjection after 30 min with systemic nicotine, for comparison (Fig. 10*A*). Notably, STR nicotine microinjection effects appeared to correlate with systemic nicotine effects in each experiment, and accordingly we visually sorted STR injection experiments into two groups: those with an apparent effect of systemic nicotine versus those without. Animals that visually showed systemic nicotine enhancement of the narrowest-notch (0.1 octave) NN-evoked response (paired *t* test, *n* = 6, *t*_5_ = 2.229, *p* = 0.076) also exhibited enhanced responses after STR microinjection (Fig. 10*B*, right; paired *t* test, *n* = 6, *t*_5_ = 3.198 *p* = 0.02). In contrast, animals with little effect of systemic nicotine (paired *t* test, *n* = 4, *t*_3_ = 1.421, *p* = 0.25) exhibited no effect of STR injection (Fig. 10*B*; paired *t* test, *n* = 4, *t*_3_ = 0.3944, *p* = 0.7). Thus, the variable effects of nicotine microinjection into STR were at least partly due to inter-animal variability in sensitivity to nicotine. Note that the lack of microinjection effect in the subset of animals that was insensitive to nicotine, despite verified injection sites within STR, also serves as a control that microinjections per se do not alter cortical responses.

The results thus far implicate auditory subcortical nuclei and the thalamocortical pathway in systemic nicotine-induced response suppression and facilitation, respectively. However, prior studies using intracortical microinjection of antagonists to block effects of systemic nicotine have suggested that both effects can arise within A1 (Kawai et al., 2011; Intskirveli and Metherate, 2012). This raises the possibility that the overall effects of systemic nicotine may depend on independent nicotinic actions in subcortical regions (nicotinic suppression), the thalamocortical pathway (enhancement) and A1 (both suppression and enhancement). As a final manipulation, therefore, we investigated the effects of local microinjection in A1. Initially, we attempted to inject nicotine itself, but were unable to obtain consistent results. We then tried a different approach, to inject a positive allosteric modulator of nAChRs, NS9283. This drug does not activate nAChRs on its own, but does amplify nicotine- or ACh-evoked responses for nAChRs containing α2 or α4 subunits (Timmermann et al., 2012). Since nicotinic effects in A1 are thought to depend on α4β2 nAChRs (Kawai et al., 2011), NS9283 should enhance effects of endogenous ACh or exogenous nicotine acting at these receptors.

**Figure 11.**
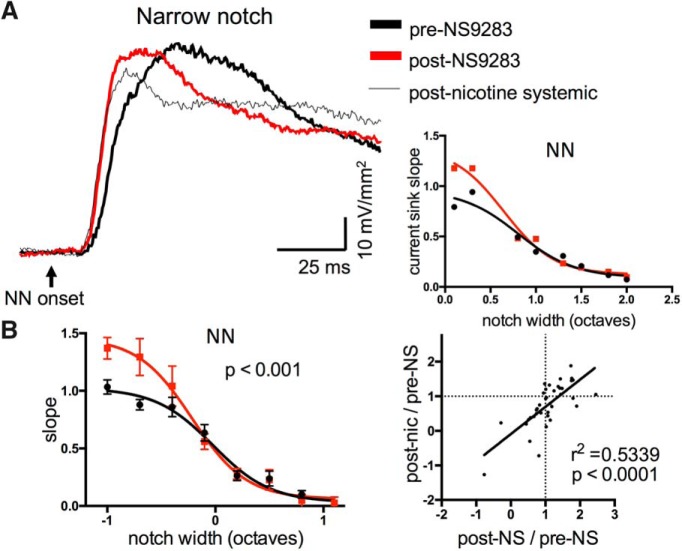
Cortical microinjection of positive allosteric modulator, NS9283, enhanced TINN-evoked responses in A1. ***A***, Example of NN-evoked current sink in A1 enhanced by intracortical microinjection of NS9283, and subsequently by systemic nicotine (left); graph (right) shows response magnitudes before and after NS9283 microinjection. ***B***, Group data (*n* = 10) of NS9283 effects on NN-evoked responses (left), implicating enhancement by endogenous ACh. Graph on right correlates effect of NS9283 microinjection (post-NS/pre-NS ratio) with that of systemic nicotine (post-nic/pre-NS ratio), all notch widths included. Correlation reflects similar effects of both drugs, including enhancement at narrow notch widths and suppression at intermediate notch widths.

Microinjection of NS9283 (10 µM, 50-100 µl) in A1 resulted in enhanced NN-evoked responses to narrow-notch stimuli, and in some cases reduced responses to intermediate-notch stimuli (Fig. 11*A*,*B*; *n* = 10, repeated-measures two-way ANOVA, main notch effect *F*_(21,45)_ = 14.2, *p* < 0.0001, main NS9283 effect *F*_(1,45)_ = 15.42, *p* = 0.003, interaction *F*_(21,45)_ = 2.896, *p* = 0.0014). Microinjection of vehicle (DMSO) had no effect (not shown, *n* = 4, *F*_(1,10)_ = 1.932, *p* = 0.19). Surprisingly, NS9283 injections had no effect on tone-evoked responses (not shown, repeated measures two-way ANOVA, *p* = 0.75). Although the main effect of NS9283 appears to be enhanced responses to narrow-notch stimuli, in individual cases we also saw reduced responses to intermediate-notch stimuli, i.e., dual effects resembling those of systemic nicotine (Fig. 2). Since we followed NS9283 microinjections with systemic nicotine after 15 min, we were able to compare directly in each animal the effects of NS9283 with any further effect of systemic nicotine. The results are in Figure 11*B*, right, which plots postdrug/predrug ratios for NS2983 (*x*-axis) vs. systemic nicotine (*y*-axis), including data for all notch widths. Notably, both drugs tended to produce similar effects, either enhancement (at narrower notches) or suppression (at intermediate notches; *r* ^2^ = 0.5339, *p* < 0.0001). These data suggest that both nicotinic enhancement and suppression of responses can arise within A1.

## Discussion

**Figure 12.**
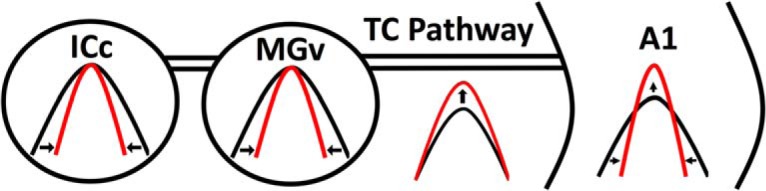
Summary schematic depicting effects of systemic nicotine on RFs in the auditory lemniscal pathway. Nicotine narrows RFs in ICc and MGv, increases gain in the thalamocortical (TC) pathway, and both narrows RFs and increases gain in A1. Nicotine effects integrate to produce the RF changes observed in A1.

We have examined the effects of systemic nicotine on auditory processing, using CSD analysis of TINN-evoked responses. In A1, systemic nicotine enhanced responses to narrow-notch NN stimuli, reduced responses to intermediate-notch stimuli, and enhanced responses to CF tones (presented either alone, or within wide-notch stimuli); these results demonstrate increased response gain within narrowed RFs. Modulation of RFs in A1 reflected nicotine effects at several levels in the auditory pathway, including response suppression that varied with notch width (narrower RFs) in ICc and MGv, facilitation in the thalamocortical pathway, and both suppression and facilitation within A1. These effects of systemic nicotine, integrated and relayed up the lemniscal auditory pathway, produce increased gain within narrowed RFs in A1 (Fig. 12, discussed below).

### Use of TINN stimuli and CSD analysis to probe auditory processing

TINN stimuli are widely used in psychoacoustics to estimate perceptual filters (Patterson, 1976). Here, we use TINN stimuli and CSD analysis to derive a novel measure of RFs, the physiologic analog of perceptual filters, with several advantages over RFs created using tone stimuli and unit recordings. TINN stimuli activate multiple frequency channels simultaneously to better approximate naturalistic stimuli, and the 50 ms stimulus onset asynchrony (NN vs tone) provides a snapshot of spectrotemporal dynamics. CSD analysis is based on LFPs, which capture subthreshold synaptic, activity, and the resulting RFs are broader than spike-based RFs (Galván et al., 2002; Noreña and Eggermont, 2002; Kaur et al., 2004). Three features emerge from this analysis (Figs. 1, 2): first, the response to NN stimulation alone (measured before presentation of the CF tone) is used to derive a RF; second, the response to the CF tone when it follows the widest-notch stimuli (i.e., NN stimulation outside the RF) is similar to that following presentation of a CF tone alone; and third, the 50 ms delay before tone presentation permits assessment of spectrotemporal processes, including response adaptation as well as feedforward and lateral inhibition. That is, the 50 ms delay is short enough so that responses are adapted when there is overlap between the neural populations excited by the NN and tone stimuli, and long enough for development of cortical IPSPs (Metherate and Ashe, 1994; Wehr and Zador, 2005). A fixed delay will not capture all temporal features; however, intermediate-width NN stimuli that stimulate the RF edges, as evidenced by weak excitation at the recording site, nonetheless produced strong reduction of tone-evoked response, likely demonstrating the presence of lateral inhibition similar to that produced by two-tone stimulus protocols (Semple, 1995). Overall, use of a TINN stimulus provides a useful snapshot of RF dynamics.

### Nicotinic modulation of responses in A1

Our conclusion that nicotine increases response gain within narrowed RFs extends findings that nicotine enhanced responses to CF stimuli and reduced responses to nonCF stimuli (one to two octaves distant from CF; Liang et al., 2008; Intskirveli and Metherate, 2012). Nicotinic enhancement of CF-evoked responses can be blocked by intracortical infusion of dihydo-β-erythroidine (DHβE), an antagonist of α4β2-containing nAChRs (Kawai et al., 2011), or by inhibition of MAP kinase activated by nAChRs (Intskirveli and Metherate, 2012). Importantly, nicotinic enhancement of inputs to L4, or subsequent intracortical activity, was prevented by inhibition of MAP kinase in the thalamocortical pathway, or A1, respectively. The present findings that microinjection of nicotine into the thalamocortical pathway enhanced the L4 current sink further support the notion that nAChRs in the auditory thalamocortical pathway enhance thalamocortical inputs (Kawai et al., 2007).

Nicotine reduced the cortical response to intermediate-notch NN stimuli, indicating a narrowed RF. This effect reflects, in part, RF narrowing in afferent pathways since similar effects were observed in ICc and MGv, and in L4 when intracortical activity was silenced by muscimol. A logical consequence of narrower RFs could be reduced adaptation following stimulation of RF edges, consistent with our observation of CF-evoked responses being less suppressed by intermediate-width stimuli (Fig. 2*C*). That is, reduced excitation in A1 following stimulation of RF edges could, in turn, reduce the adaptation of tone-evoked responses. However, we cannot distinguish between reduced suppression and overt facilitation of tone-evoked responses, especially since the latter is apparent with wide-notch stimuli as well as stimulation with CF tones presented alone (Fig. 2*C*); either or both mechanisms would enhance response magnitude, and may contribute to the effect observed with intermediate-notch stimuli. Moreover, since reduction of cortical responses may involve an intracortical mechanism (effects of NS9283), narrowing of RFs could result from enhanced intracortical inhibition. In visual cortex, lateral inhibition has been attributed to GABAergic interneurons that express somatostatin (SOM; Adesnik et al., 2013), and SOM interneurons are excited by nicotine (Jia et al., 2009; Leão et al., 2012). Alternatively, parvalbumin (PV)-expressing interneurons are implicated in feedforward and lateral inhibition (PV neurons have broader RFs than excitatory neurons to which they project; Wu et al., 2008), and PV interneurons are excited by nicotine in some studies (Poorthuis et al., 2013), but not others (Porter et al., 1999). Thus, nicotinic enhancement of PV-interneurons may enhance both kinds of inhibition. Other interneurons expressing vasoactive intestinal peptide (VIP) may contribute to the facilitatory effects of nicotine via disinhibition, e.g., inhibition of PV interneurons (Porter et al., 1999; Alitto and Dan, 2013; Fu et al., 2014; Bell et al., 2015). Thus, multiple nicotinic mechanisms may contribute to narrowing of RFs, reduced suppression and/or overt facilitation of tone-evoked responses in A1.

### Nicotine effects integrate across levels of the ascending auditory pathway

Our hypothesis that effects of systemic nicotine originate largely in cortex was refuted by silencing cortex using muscimol. At the dose employed, intracortical activity was largely silenced, but the remaining activity, monosynaptic thalamocortical input (Intskirveli et al., 2016), clearly exhibited increased gain and narrowed RFs after systemic nicotine. These effects arose from different loci in the auditory pathway, including narrowed RFs in ICc and MGv, and increased gain in the thalamocortical pathway. Note that the exact nicotinic actions responsible for response suppression (narrowed RFs) in ICc and MGv are not known, though both structures exhibit a high density of nAChRs (Morley and Happe, 2000; Bieszczad et al., 2012). Also, RF narrowing in ICc and MGv was not associated with subcortical facilitation, which occurred only after nicotine microinjection into the thalamocortical pathway, demonstrating the locus of enhanced cortical inputs . This finding is consistent with increased excitability of thalamocortical axons and the presence of nAChRs in the thalamocortical white matter (Ding et al., 2004; Kawai et al., 2007; Bieszczad et al., 2012). Finally, nAChR-mediated suppression and facilitation also occur within A1, as demonstrated by effects of NS9283, as well as the effects of intracortical DHβE in previous studies (Kawai et al., 2011; Intskirveli and Metherate, 2012). Importantly, since NS9283 is a positive allosteric modulator, its effects imply similar actions of endogenous ACh.

Figure 12 summarizes our main findings, using a framework for understanding the effects of systemic nicotine on auditory processing. Nicotinic effects in ICc and MGv are solely suppressive, yet vary with spectral distance from CF to narrow RFs, whereas effects in the thalamocortical pathway are solely facilitatory. Suppression and facilitation also occur within A1, and the integrated effects of systemic nicotine produce increased gain within narrowed RFs. Although nAChRs gate excitatory currents, suppressive effects of nicotine occur widely due to nAChRs located on inhibitory neuron somata to cause overt excitation, or on presynaptic terminals to enhance GABA release (Wonnacott, 1997; Albuquerque et al., 2009). As described above, the facilitatory effect of nicotine in the thalamocortical pathway likely results from increased axon excitability, and facilitatory effects within A1 may arise from excitation or disinhibition. Detailed cellular analyses in each region will be needed to understand these actions, but the strength of the CSD approach is to reveal the overall effect in each region.

### Relevance of results to auditory-cognitive function

An important question is to what extent the results relate to auditory-cognitive function, given the anesthetized preparation. Anesthesia permits a relatively stable brain state, and urethane specifically does not depress nAChRs (Materials and Methods). Evoked responses in the anesthetized auditory cortex resemble responses in some, but not all, waking states (e.g., passive, aroused, or attentive; Clementz et al., 2002; Kato et al., 2015; Reinhold et al., 2015). The nicotinic increased gain observed here resembles that seen for some sensory-evoked responses in awake animals and nonsmoking humans (wearing a nicotine patch; Guha and Pradhan, 1972; Bringmann, 1994; Harkrider and Champlin, 2001). Thus, the effects are likely relevant for some, but not all, waking states in humans.

Intriguingly, the main effects of systemic nicotine, increased gain within narrowed RFs, also occur during auditory attention in humans and nonhuman primates (Okamoto et al., 2007; Lakatos et al., 2013; O’Connell et al., 2014). These effects may underlie the dual perceptual consequences of nicotine, i.e., increased processing capacity and narrowed attention (Friedman et al., 1974; Kassel, 1997; Knott et al., 2011). The similarity of effects (nicotine vs attention) may reflect the involvement of the cholinergic system in attention (Levin and Mcclernon, 2006; Albuquerque et al., 2009; Hasselmo and Sarter, 2011; Miwa et al., 2011). Consequently, the findings also suggest the possible therapeutic use of nicotine to treat disorders involving diminished attention, which are increasingly being recognized as a subset of central auditory processing disorders (Moore, 2015).

## Synthesis

Reviewing Editor: Darcy Kelley, Columbia University Decisions are customarily a result of the Reviewing Editor and the peer reviewers coming together and discussing their recommendations until a consensus is reached. When revisions are invited, a fact-based synthesis statement explaining their decision and outlining what is needed to prepare a revision will be listed below. Note: If this manuscript was transferred from JNeurosci and a decision was made to accept the manuscript without peer review, a brief statement to this effect will instead be what is listed below.

This manuscript was reviewed and re-reviewed at J. Neurosci. previously and then forwarded to eNeuro for consideration.

The authors use a current source density and tone in notched noise to probe the effects of nicotine on auditory receptive fields in cortex (A1) and subcortical regions in anesthetized mice. They have substantively addressed the issues raised by the previous reviewers and include a rationale for the utility of using anesthetized mice versus mice in various awake states (uniformity). The results directly address the issue of mechanisms for cognitive effects of nicotine, the studies are well conducted and the results should be of interest to the readership of eNeuro.
